# Prevalence, Serotype Distribution, and Risk Factors Associated With *Streptococcus pneumoniae* Carriage in Older Adults in Denmark, 2019–2022

**DOI:** 10.1093/ofid/ofag452

**Published:** 2026-07-24

**Authors:** Michaela Tinggaard, Hans-Christian Slotved, Randi Føns Petersen, Nichlas Hovmand, Thomas Benfield

**Affiliations:** Department of Infectious Diseases, Copenhagen University Hospital—Amager and Hvidovre, Hvidovre, Denmark; Department of Bacteria, Parasites and Fungi, Statens Serum Institut, Copenhagen, Denmark; Department of Natural and Environmental Science, Roskilde University, Roskilde, Denmark; Department of Bacteria, Parasites and Fungi, Statens Serum Institut, Copenhagen, Denmark; Department of Infectious Diseases, Copenhagen University Hospital—Amager and Hvidovre, Hvidovre, Denmark; Department of Infectious Diseases, Copenhagen University Hospital—Amager and Hvidovre, Hvidovre, Denmark; Department of Clinical Medicine, Faculty of Health and Medical Sciences, University of Copenhagen, Copenhagen, Denmark

**Keywords:** adults, pneumococcal carriage, pneumococcal invasive disease, pneumococcal vaccine, serotypes

## Abstract

**Background:**

The burden of pneumococcal disease in older adults remains high in the postpneumococcal conjugate vaccine (PCV) era. We hypothesized that older adults may represent a carriage reservoir of *Streptococcus pneumoniae*.

**Methods:**

Paired naso- and oropharyngeal (NP and OP) swabs and questionnaires were collected during 2019–2022 from adults ≥65 years of age. Pneumococcal carriage and serotype distribution were determined by phenotypical and molecular methods.

**Results:**

A total of 172/1764 participants (9.8% [95% confidence interval {CI} 8.4–11.2]) were positive for carriage of *S. pneumoniae* by either culture (0.3%) or *lytA/piaB* DNA in NP (2.0%) or OP (8.4%) samples. Overall, 118 serogroup- or serotype-specific carriage events were identified by multiplex polymerase chain reaction across 16 distinct serospecificities in 93 samples, while 79 samples were nontypeable. PCV13 types accounted for 8.0%, nonvaccine types for 11.0% and polysaccharide pneumococcal vaccine 23 (PPV23)/non-PCV13 types for 81.0%. All carriage serotypes, except serotype 2, had been identified in invasive pneumococcal disease (IPD) isolates among adults ≥65 years during 2019–2022. Having young grandchildren was associated with increased odds of carriage (adjusted odds ratio [OR] 1.8, 95% CI 1.2–2.5, *P* = .0031). No association between PPV23 immunization and carriage was observed (OR 0.8, 95% CI .5–1.3).

**Conclusions:**

Older adults were carriers of a wide range of IPD-causing serotypes. Both carriage and IPD serotypes were mainly comprised of non-PCV13 serotypes. Immunization with higher valency conjugated vaccines is likely required to reduce residual pneumococcal disease among older adults.

## BACKGROUND

*Streptococcus pneumoniae* is an important pathogen as well as a commensal bacterium of the upper respiratory tract. More than 100 pneumococcal capsular serotypes are known [1, 2]. Serotypes vary in their ability to colonize their host and to cause invasive disease [3, 4]. Surveillance of the prevalence and distribution of pneumococcal carriage serotypes is a prerequisite to guide public health measures and to identify emerging disease-causing serotypes.

Invasive pneumococcal disease (IPD) incidence peaks among young children and older adults, and children are considered to be the major source of transmission in the population [5].

In 2007, the 7-valent pneumococcal conjugate vaccine (PCV7) was introduced in the Danish childhood vaccination program and administered at ages 3, 5, and 12 months. In 2010, the PCV7 was replaced by the PCV13, which again was replaced by the PCV15 in 2025. The introduction of PCVs led to a reduction in IPD incidence and mortality among infants and toddlers [6]. Similarly, a reduction in incidence and mortality in older adults was shown, likely due to herd protection. National surveillance data indicate that coverage of the Danish childhood immunization program remains high, above 95% [7].

Pneumococcal vaccines have only been offered to adults in Denmark on a risk assessment basis, except during the COVID-19 pandemic, at which time the pneumococcal polysaccharide vaccine (PPV23) was offered free of charge for all adults above 65 years of age. Nevertheless, substantial residual disease among older adults remains due to vaccine types (VTs) and, to a greater extent, non-VTs (NVTs) [8] as well as by serotypes infrequently carried by children [9]. This raises the question of whether adults constitute a pneumococcal reservoir of their own and of which serotypes colonize adults.

Our objective was to estimate the prevalence and associated risk factors for pneumococcal carriage among adults above 65 years of age. Further, to estimate the distribution of pneumococcal carriage serotypes and compare these to contemporary invasive disease serotypes.

## METHODS

### Carriage

#### Study Design and Data Collection

We conducted a cross-sectional pneumococcal carriage study, “P3,” among adults above 64 years of age (target 1500) residing in the Copenhagen area from January 2019 to November 2022. Eligible participants were invited to participate by their general practitioner at 19 practices and a COVID-19 vaccination center. Individuals were ineligible if they had symptoms of upper respiratory tract infection or had received antibiotics in the past month.

A nasopharyngeal (NP) swab, an oropharyngeal (OP) swab, and a questionnaire with information on morbidity, smoking status, antibiotic use, grandchildren and recent travel history were collected. Swabs were transferred to transport media (1 mL Amies liquid, ESwab®, Copan, Italy) and transported to the Statens Serum Institute (SSI) within 24 hours, where they were transferred to cryotubes containing 110 µL 10% glycerol and stored at −80°C.

Pneumococcal vaccination status of participants was retrieved from The Danish Vaccination Registry [10].

Data from 2019 to 2021 on pneumococcal carriage have been reported previously [11], whereas the data from 2022 as well as data on serotyping were not included in that publication.

#### Identification of *S. pneumoniae*

Samples were analyzed by phenotypical and molecular methods.

Samples were thawed at room temperature, and 15 µL was incubated with 3 mL of serum broth for 24 hours at 35–36 C°. A rapid latex agglutination test (ImmuLex™ *S. pneumoniae* Omni kit, SSI Diagnostica, Hillerød, Denmark) was performed if turbidity or precipitation was present. Then, 10 µL of an Omni-positive sample was plated on 10% blood agar, incubated overnight at 35–36 C° and evaluated by optochin susceptibility. Colonies were further analyzed by matrix-associated laser desorption/ionization time of flight mass spectrometry [12]. Samples with ambiguous readings were re-cultured.

Nucleic acids were extracted from 200 µL of thawed sample material by 20% Chelex solution (Bio-Rad Laboratories AB, Denmark) according to the manufacturer's instructions and stored at −20° C. Identification of pneumococcal DNA was initially performed using a *lytA* targeted real-time quantitative polymerase chain reaction (qPCR) analysis [13]. Samples with a *lytA* signal were broth enriched by transferring 15 µL of sample solution to 3 mL of serum broth and stored overnight at 35–36 °C, and 250 µL was preincubated with 250 µL MagNA Pure 96 External Lysis buffer for 10 minutes at 65 °C. DNA was extracted with the MagNA Pure 96 DNA and Viral NA Large Volume Kit (Roche Diagnostics, Mannheim, Germany). A second qPCR was then performed using primers and probes specific for *lytA* and *piaB* [14]. Samples were considered positive for *S. pneumoniae* if cycle threshold (Ct) values were below 40 for both target genes.

#### Serotyping of *S. pneumoniae*

Culture positive samples were serotyped by latex agglutination (ImmuLex™ Pneumotest kit, SSI Diagnostica) combined with the Neufeld capsular swelling (Quellung) reaction using type-specific pneumococcal rabbit antisera.

Broth-enriched samples positive by *lytA/piaB* qPCR were serotyped using the protocol for multiplex conventional PCR (mc-PCR) from the Streptococcus Laboratory, The Center for Disease Control [13]. Our approach used 8 reactions to identify 40 serospecificities (Supplementary Table 1), determined from their frequency in IPD isolates in Denmark and inclusion in the PCV13 and PPV23 vaccines. Certain assays targeted more than one serotype, and it was therefore not possible to distinguish 6A/6B, 6C/6D, 7C/7B/40, 7F/7A, 9N/9L, 9V/9A, 10F/10C/33C, 11A/11D, 12F/12A/44/46, 15A/15F, 15B/15C, 18A/18B/18C/18F, 22F/22A, 24A/24B/24F, 33F/33A/37, 35A/35C/42, 35F/47F, and 38/25F/25A.

Fifty l*ytA/piaB*-negative samples (25 OP and 25 NP samples, respectively) were serotyped as (nonpneumococcal) controls of specificity. Serotypes detected in these controls were excluded from further analyses when identified in *lytA/piaB*-positive samples, as they could not be confidently attributed to pneumococci and may originate from nonpneumococcal DNA.

As a post hoc analysis, we performed a *t*-test to compare *lytA* Ct values between typed and nontyped samples.

All laboratory analyses were performed at SSI.

### IPD Surveillance

Information on IPD during 2010–2022 was obtained from the Danish laboratory surveillance system based at the Neisseria and Streptococcus Reference Laboratory, SSI, which contains laboratory-confirmed data on IPD, defined as the detection of *S. pneumoniae* from blood, cerebrospinal fluid, or other normally sterile sites. From October 2007, it became mandatory to submit invasive pneumococcal isolates from all departments of clinical microbiology as part of the enhanced surveillance of IPD following the implementation of PCVs in the childhood immunization program [11].

### Statistical Analysis

Categorical values are presented as counts (percentages) and continuous variables as medians (interquartile ranges [IQRs]). The prevalence of *S. pneumoniae* carriage was defined as the number of participants with at least one NP or OP positive sample by culture or qPCR divided by the total study population. Prevalence is presented as percentages with 95% confidence interval (CI).

Univariable and multivariable logistic regression models were conducted with carriage versus noncarriage as outcome. Independent variables were selected a priori. Participants with missing values on variables included in the analyses were excluded from the models. Crude odds ratios (ORs) with 95% CI were adjusted for potentially relevant confounders that were predefined separately for each variable of interest.

Incidence rates (IRs) of VT-specific IPD (PCV13 type, PPV23/non-PCV13 type, and NVT) among adults above 65 years of age were calculated by year (2010–2022). The IRs were computed as the number of IPD cases per 100.000 person-years at risk.

As post hoc analyses, we compared the proportion of PPV23 serotype carriers among PPV23 vaccinated versus unvaccinated by χ^2^-test. The significance threshold was set at 0.05.

All statistical analyses were performed using R Software version 4.1.3 (R Foundation for Statistical Computing, Vienna, Austria).

## RESULTS

### Study Population Characteristics

A total of 1764 adults were included in the study. From these, 1869 paired OP and NP samples were provided, as 105 participants contributed 2 sets of samples during the 4 years of inclusion. Only the first set of samples (n = 1764) was included in the primary analysis. Of these, questionnaires were available for 1670 (94.7%) of participants.

Our study population consisted of 54% females and had a median age of 74 years (IQR 70–79) (Table 1). Sixteen percent (n = 234) were active smokers, 40% (n = 590) had had pneumonia at any time prior to sampling, and 12% (n = 192) had received antibiotics within 6 months of sampling. Fifty-five percent (n = 817) had one or more comorbidity, 43% (n = 627) had received PPV23 prior to study inclusion (mean time 192 days before sampling), and 8% (n = 120) had been vaccinated with both PCV13 and PPV23. Finally, 20% (n = 298) had grandchildren under the age of 5. A total of 511 participants were sampled before (January 2019 to 11 March 2020) the COVID-19 lockdown in Denmark, 240 were sampled during lockdown (12 March 2020 to February 2021), and 907 were sampled after the withdrawal of containment measures (March 2021 to November 2022).

**Table 1.** Characteristics of Adult Participants (N = 1670) Enrolled in the Study

	Total N = 1670	Noncarriage N = 1506	Carriage N = 164	Single-Serotype Carriage^d^ N = 146	Multiple-Serotype Carriage N = 18
**Age, median [IQR]**	74 (70–79)	74 (70–79)	73 (69–78)	73 (69–79)	75 (71–77)
64–69	449 (27)	397 (27)	52 (32)	47 (32)	5 (28)
70–74	463 (28)	413 (28)	50 (30)	45 (31)	5 (28)
75–79	405 (24)	376 (25)	29 (18)	25 (17)	4 (22)
>80	341 (21)	308 (20)	33 (20)	29 (20)	4 (22)
Missing	12	12	…	…	…
**Sex**					
Male	764 (46)	688 (46)	76 (46)	68 (47)	8 (44)
Female	906 (54)	818 (54)	88 (54)	78 (53)	10 (56)
**Period of sampling**					
Prelockdown**^a^**	511 (31)	445 (30)	66 (40)	59 (40)	7 (39)
Lockdown**^b^**	240 (14)	230 (15)	10 (6)	9 (6)	1 (6)
Postlockdown**^c^**	907 (55)	819 (55)	88 (54)	78 (54)	10 (56)
Missing	12	12	…	…	…
**Current smoking status**					
Nonsmoker	1408 (85)	1267 (84)	141 (86)	125 (86)	16 (89)
Smoker	255 (15)	233 (16)	22 (14)	20 (14)	2 (11)
Missing	7	6	1	1	…
**Comorbidity**					
0	715 (43)	635 (42)	80 (49)	71 (49)	9 (50)
≥1	946 (57)	864 (58)	82 (51)	73 (51)	9 (50)
Missing	9	7	2	2	…
**Prior pneumonia**					
No	1008 (60)	898 (60)	110 (68)	98 (68)	12 (67)
Yes	657 (40)	604 (40)	53 (32)	47 (32)	6 (33)
Missing	5	4	1	1	…
**Antibiotics past 6 m**					
No	1462 (88)	1311 (88)	151 (93)	134 (93)	17 (94)
Yes	198 (12)	187 (12)	11 (7)	10 (7)	1 (6)
Missing	10	8	2	2	…
**Pneumococcal vaccination status**					
None	730 (44)	644 (43)	86 (52)	76 (52)	10 (56)
PCV13	21 (1)	18 (1)	3 (2)	3 (2)	0 (0)
PPV23	762 (46)	695 (47)	67 (41)	60 (41)	7 (39)
PCV13 + PPV23	143 (9)	135 (9)	8 (5)	7 (5)	1 (6)
Missing	14	14	…	…	…
**Grandchildren** <**5 y/o**					
No	1298 (78)	1189 (79)	109 (67)	99 (68)	10 (56)
Yes	372 (22)	317 (21)	55 (33)	47 (32)	8 (44)
**Travel abroad past 6 m**					
No	1152 (69)	1044 (70)	108 (66)	94 (64)	14 (78)
Yes	514 (31)	458 (30)	56 (34)	52 (36)	4 (22)
Missing	4	4	…	…	…

Abbreviation: IQR, interquartile range.

^a^January 2019 to 11 March 2020.

^b^12 March 2020 to February 2021.

^c^March 2021 to November 2022.

^d^Including nontypeables.

### Prevalence and Serotype Distribution of Pneumococcal Carriage

Among the 1764 participants included, 4 (0.3%) samples were culture positive, and 172 were positive by *lytA* and *piaB* qPCR in either an NP sample (N = 34 [2.0%]), an OP sample (n = 147 [8.4%]), or both (n = 9 [0.5%]). This corresponds to an overall pneumococcal carriage prevalence of 172/1764 = 9.8% (95% CI 8.4–11.2). Ct values for *lytA* and *piaB* in NP and OP samples, respectively, can be seen in Supplementary Figure 1 [11].

**Figure 1.**
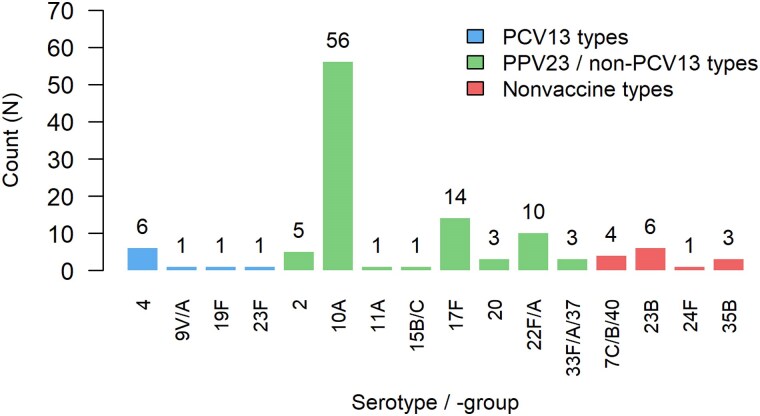
Distribution of serogroups/types of pneumococci in samples from 1764 adults 65 y or older in Denmark, 2019–2022.

Of the 172 individuals with *lytA/piaB*-positive samples, one or more serotypes were identified in 93 samples, while no serotype was detected in the remaining 79 samples, referred to as “nontypeables.” Two serotypes were identified in 14 participants and 3 types in 5 participants, while the remaining participants carried a single serotype. A serotype/group was assigned to a total of 118 samples within 16 different serospecificities in either an NP or OP sample (Figure 1). Of these, 12 were identified in NP samples and 106 in OP samples. The most common serotype in both NP and OP samples was 10A (n = 56). The majority were PPV23/non-PCV13 types (81%, n = 93) followed by NVTs (11%, n = 14) and PCV13 types (8%, n = 9).

A serotype was identified in 3 of the 4 culture positive samples: 10A, 22F, and 24F, respectively. These serotypes were confirmed by mc-PCR.

Among the 50 *lytA/piaB*-negative control samples, no serotypes were detected in NP specimens, but OP samples yielded detections of serotypes 5 (n = 1), 9N/9L (n = 1), 12F/12A/44/46 (n = 1), 18A/18B/18C/18D (n = 2), 24A/24B/24F (n = 3), and 39 (n = 1). These findings were considered likely false positives attributable to nonpneumococcal DNA. Within the *lytA/piaB*-positive samples, these “serotypes” were detected at the following frequencies: serogroup 24A/24B/24F (n = 102), serotype 39 (n = 22), serogroup 12F/12A/44/46 (n = 16), serogroup 18A/18B/18C/18D (n = 11), and serotype 5 (n = 8) (Supplementary Figure 2). Accordingly, they were excluded from the final results.

Finally, a *t*-test showed that nontypeable samples had significantly higher *lytA* Ct values than typeable samples (mean 32.5 vs 30.3, *P* = .002), indicating that failure to assign a serotype was related to lower pneumococcal density.

### Participants Swabbed Twice

The 105 participants contributing with OP and NP swabs twice during the study period were sampled ∼12 months apart. Three of these participants carried pneumococci at first sampling (nontypeable) and 6 at the second sampling (serotypes 4, 10A, and17F). None carried pneumococci at both sampling events. Including these samples yielded a sample carriage rate of 181/1869 = 9.7% (95% CI 8.4–11.1).

### Serotype Distribution of IPD

**Figure 2:**
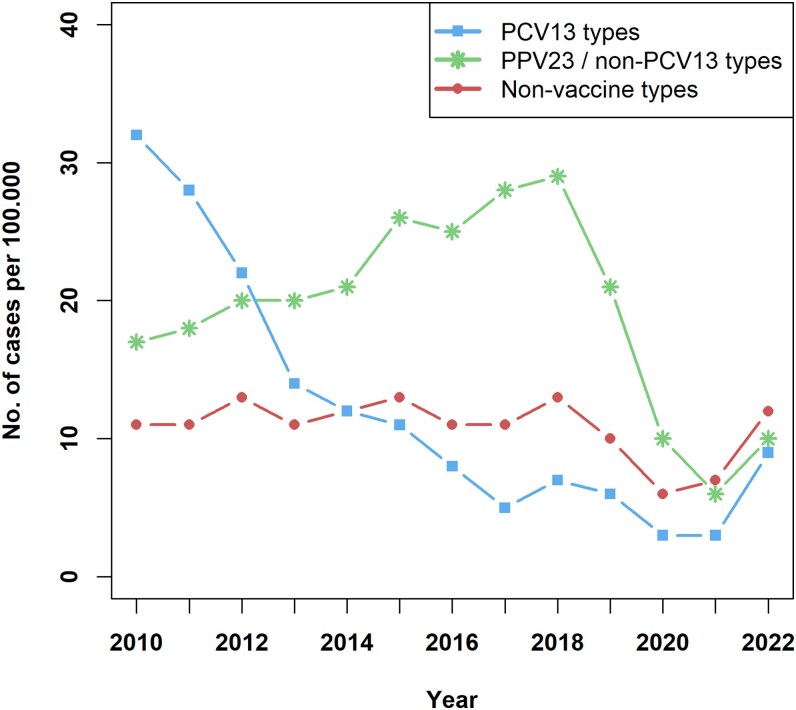
. Incidence rates of vaccine/nonvaccine type invasive pneumococcal disease during 2010–2022 in adults >65 y.

A total of 5747 cases of IPD were reported during 2010–2022 in adults 65 years or older. Of these, 5435 (94.6%) isolates were serotyped. PPV23/non-PCV13 type IPD was most frequent among older adults in the years 2013–2017 but declined during the years 2018–2021 (Figure 2). PCV13 type IPD declined during 2010–2021, while NVT IPD remained at an almost steady level. Both VT and NVT IPD increased from 2020/2021 to 2022. The most frequent IPD-causing serotypes with more than 40 cases among adults 65 years or older during 2019–2022 were 3, 8, 22F, 12F, 9N, 7C, 35F, and 24F, in descending order (Table 2).

**Table 2.** Serotype Distribution of Carriage and Invasive Pneumococcal Disease Among Adults 65 Years or Older in Denmark 2019–2022

Carriage		Invasive Pneumococcal Disease		Vaccination Coverage by PCV13, PCV15, PCV20, PCV21, or PPV23
Serogroup/type	N = 118	Serotype	N = 1341	
10A	56	10A	40	PCV20/PCV21/PPV23
Nontypeable	79	Nontypeable	2	…
17F	14	17F	30	PCV21/PPV23
22F/22A	10	22F	138	PCV15/PCV20/PCV21/PPV23
4	6	4	10	PCV13/PCV15/PCV20/PPV23
23B	6	23B	69	PCV21
2	5	2	…	PPV23
7C/7B/40	4	7B 7C	2 43	…
20	3	20	29	PCV21/PPV23
33F	3	33F	39	PCV15/PCV20/PCV21/PPV23
35B	3	35B	40	PCV21
24F	1	24F	41	PCV21
9V/9A	1	9V 9A	1 1	PCV13/PCV15/PCV20/PPV23
19F	1	19F	25	PCV13/PCV15/PCV20/PPV23
23F	1	23F	2	PCV13/PCV15/PCV20/PPV23
11A	1	11A	40	PCV20/PCV21/PPV23
15B/15C	1	15B 15C	33 27	PCV20/PPV23
8	…	8	205	PCV20/PCV21/PPV23
3	…	3	169	PCV13/PCV15/PCV20/PCV21/PPV23
12F**^a^**	…	12F	76	PCV20/PCV21/PPV23
9N/9L**^a^**	…	9N	72	PCV21/PPV23
35F	…	35F	41	…
15A	…	15A	40	PCV21
16F	…	16F	35	PCV21
23A	…	23A	26	PCV21
38	…	38	11	…
34	…	34	7	…
18A/18B/18C/18F**^a^**	…	18C	4	PCV13/PCV15/PCV20/PPV23
6A	…	6A	3	PCV13/PCV15/PCV20/PCV21/PPV23
35A	…	35A	2	…
7F	…	7F	2	PCV13/PCV15/PCV20/PCV21/PPV23
6B	…	6B	1	PCV13/PCV15/PCV20/PPV23
39**^a^**	…	39	…	…
5**^a^**	…	5	…	PCV13/PCV15/PCV20/PPV23
31**^b^**	…	31	22	PCV21
24A/24B/24F**^b^**	102	24A 24B	3 1	…
37**^b^**	…	37	3	…
35D**^b^**	…	35D	2	…
21**^b^**	…	21	2	…
28A**^b^**	…	28A	1	…
33A**^b^**	…	33A	1	…

Abbreviations: PCR, polymerase chain reaction; PCV, pneumococcal conjugate vaccine; PPV, polysaccharide pneumococcal vaccine.

^a^Excluded from *lytA/piaB*-positive samples as they were also detected in *lytA/piaB*-negative controls.

^b^Not covered by the conventional multiplex PCR used.

Except for serotype 2, all carriage types during our study period of 2019–2021 had been identified in IPD isolates among adults ≥65 years during 2019–2022. Conversely, we did not identify serotypes 3 and 8 in carriage, although these 2 serotypes made up 28% of IPD cases (n = 374/1341) among older adults during 2019–2022.

Finally, the following serotypes were identified in IPD isolates but not covered by our mc-PCR: 31, 37, 21, 35D, 28A, and 33A.

### Factors Associated With Colonization

**Figure 3:**
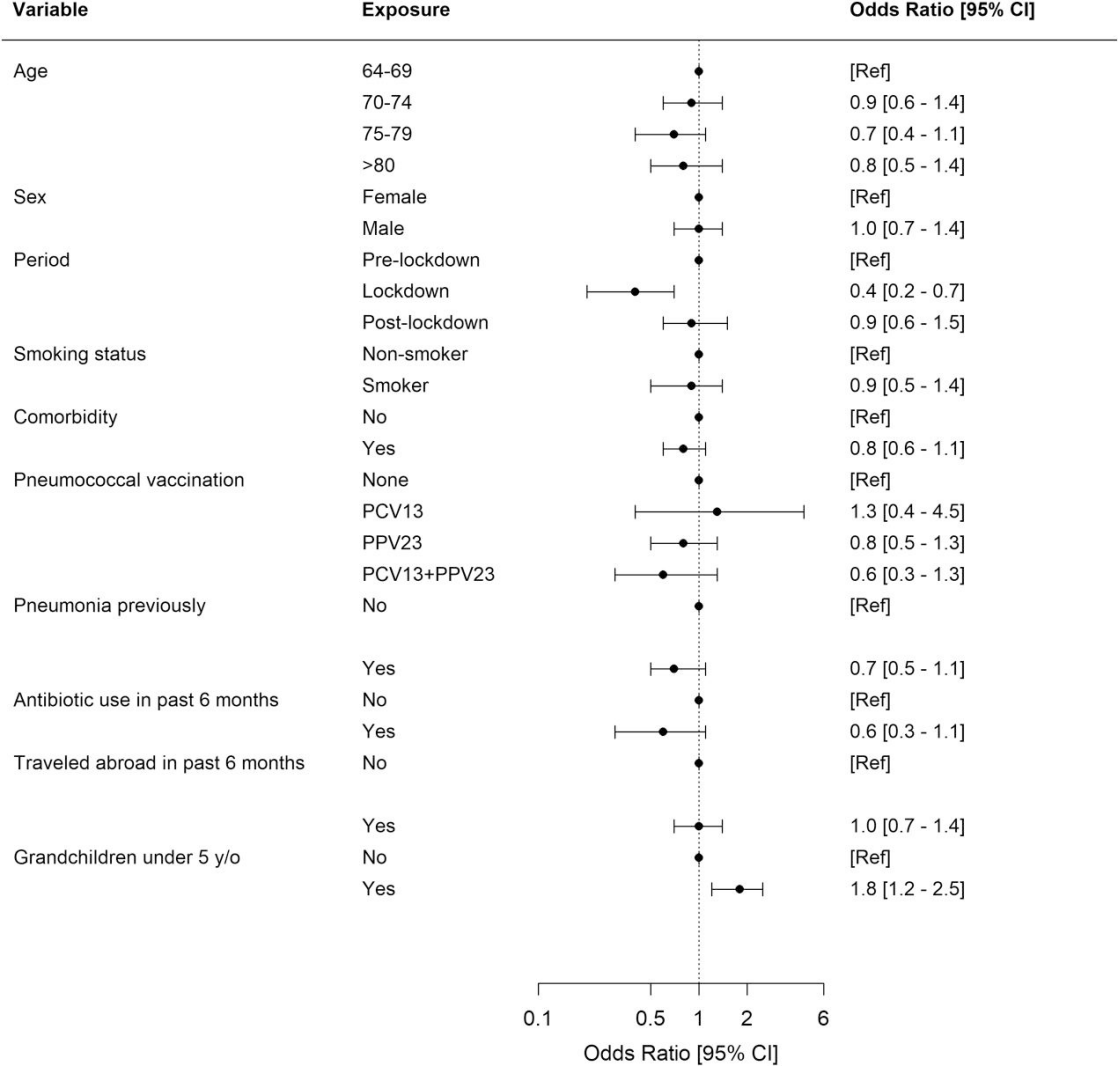
. Multivariate analysis of factors associated with pneumococcal carriage among 1628 adults.

We had complete case data in 1628 of 1670 questionnaires (97.5%) for multivariate analysis. The odds of carriage were significantly decreased among participants swabbed during the COVID-19 lockdown period in Denmark from 11 March 2020, to February 2021 (adjusted OR 0.4, 95% CI .2–.7, *P* = .0042) compared to pre- and postlockdown (Figure 3; Supplementary Table 2) as previously reported [11]. Further, having grandchildren under the age of 5 years old increased odds of pneumococcal carriage (adjusted OR 1.8, 95% CI 1.2–2.5, *P* = .0031). When carriage by study period was stratified by having grandchildren under 5 years of age (yes/no), the association between being swabbed during lockdown and decreased odds of carriage was present among participants without young grandchildren (OR 0.2, 95% CI .1–.5, *P* = .0006), but not among those with young grandchildren (OR 0.7, 95% CI .3–2.0, *P* = .58).

The proportion of PPV23 serotype carriers among PPV23 vaccinated versus unvaccinated was similar (*P* = .82).

Age, smoking, comorbidity, vaccination status, having had prior pneumonia, or receipt of antimicrobials within the past 6 months did not affect carriage in the adjusted analyses.

## DISCUSSION

We conducted the first carriage study among older adults in Denmark and found an overall carriage rate of 9.8%. Thus, older adults seem to carry pneumococci at lower frequency than children but nonetheless have a potential reservoir. Further, trends in the proportion of carried pneumococci were reflected in IPD incidence in recent years with non-PCV13 types dominating in both.

Contact with children is a known risk factor for pneumococcal carriage among adults [15–18]. Our study confirms this finding. Our observed association between being swabbed during the COVID-19 lockdown in Denmark and decreased odds of carriage was only present in participants without grandchildren. This could be due to grandparents being exposed to pneumococci more frequently during the pandemic than adults without young grandchildren. We also found that recent antibiotic use was associated with decreased odds of carriage, as expected [18, 19], although statistically insignificant.

The Danish health authorities initiated a recommendation of PPV23 immunization for adults 65 years or older in June 2020 [20]. The PPV23 national immunization program has recently been shown to have reduced PPV23 IPD by 58% among those vaccinated with PPV23 [21]. Although half of our study population had received PPV23 vaccination prior to sampling, carriage rates were comparable between PPV23 immunized and nonimmunized individuals. Further, we found no significant difference in carriage of PPV23 serotypes between vaccinated and unvaccinated individuals. Our results therefore support previous findings that PPV23 vaccination does not influence pneumococcal colonization [22]. As the number of PCV13 vaccinated individuals was low (1.0%), we lacked statistical power to assess a potential effect on carriage.

The range of serogroups and types of pneumococci in swab samples was diverse. Carriage studies including adults in the post-PCV13 period have found similar serotypes such as 10A, 22F, and 23B [23–25], but also numerous others in various frequencies [15, 26, 27]. Comparisons of carriage prevalence and serotype circulation between different countries are, however, complicated by differences in sampling sites, detection and serotyping methodology, immunization programs, and selective pressure. Nevertheless, our prevalence estimate agrees well with the overall carriage estimate of 9.0% from a meta-analysis on pneumococcal carriage among adults above 60 years of age [28]. Culture identification of *S. pneumoniae* yielded very few positive samples in our study, but molecular methods increased detection significantly. The density of pneumococci in the pharynx appears to decrease with age [29], which could explain the low detection rate by culture. This agrees with other studies finding lower carriage prevalence in adults when applying conventional methods [12, 25, 27] compared with molecular methods [14, 16, 17, 19, 23, 26, 30–32].

Most of the serotypes identified in carriage in older adults were also a cause of IPD during the study period, but in varying degrees. A third of the more than 100 different known serotypes of pneumococci are considered to be invasive types [17]. Based on their abundance in IPD relative to carriage, serotypes with a high invasive potential include, but are not limited to 1, 3, 7F, 8, 9N, 12F, and 22F [15, 33–36]. These estimates are primarily based on data from children. In our study population, serotypes carried in low frequency or absent in carriage, yet accounting for a relatively high proportion of IPD, included 3, 8, 7C, 15B/C, 17F, 19F, 20, 22F, 23B, 33F, and 35B—suggesting a relatively high disease potential. Most of these serotypes are covered by PPV23 (except 7C and 23B) and by new higher valency conjugate vaccines (Table 2). Conversely, serotype 2 was detected in carriage, but did not account for any IPD cases in recent years, indicating low invasive potential of this serotype. Serotype 10A was clearly the most frequently carried serotype in our study population and contributed to the IPD burden in the same time period. Serotype 10A has not been covered by PCV13/15 but is included in PPV23 and the higher valency PCVs.

Serotypes 3 and 8 constituted nearly a third of the total amount of IPD cases among older adults in 2019–2022. Even so, we did not find any serotype 3 or 8 in the swabs from our carriage study population. Some serotype 8 isolates have been shown to lack the *piaB* gene [37] and may therefore be underestimated in this study. Serotype 3 has been reported to be a poor colonizer but associated with high morbidity and mortality [38]. The rise in serotype 3 cases in Europe in recent years has been a subject of concern as it may reflect a diminished herd effect among adults from PCV13 use in infants [8, 35, 39]. PCV13 provides poor protection because of the capacity of serotype 3 to evade opsonization by anticapsular antibodies [40]. Also, PCV13 does not seem to affect carriage of serotype 3 in children [41]. Transmission from children to adults may therefore remain high, although we found no evidence that older adults contain a serotype 3 reservoir of their own.

Serogroup 24A/24B/24F was detected in high frequency in the *lytA/piaB*-positive samples but were excluded from the results as this serogroup was also detected in the pneumococcal negative controls. It has indeed been demonstrated that nonpneumococcal oral streptococci (eg, *Streptococcus mitis* and *Streptococcus oralis*) can harbor pneumococcus-like capsular polysaccharide (cps) loci, including sequences closely related to serotype 24F [42]. However, serotype 24F was isolated from 1 of the 4 culture positive samples in our study, which is unlikely to be a false positive finding [43]. Some of the serogroup 24A/24B/24F detected in our pneumococcal-positive samples could therefore be true positives. Serotype 24F was one of the most frequent serotypes causing NVT IPD in Danish adults above 65 years of age in 2012, 2014–15, and 2022 and may indeed be circulating in the population. Serotype 24F is not covered by PCV13, PCV20, or PPV23 but is included in the novel 21-valent PCV [44].

The strengths of this study include the high number of participants, all of which contributed to paired OP and NP samples. Further, our study provided longitudinal data over a 3-year study period. Limitations include the lack of household density and the degree of contact between the participants and their grandchildren or other children. Further, we have no information on incidence and serotype patterns in noninvasive pneumococcal disease that also may direct immunization programs.

Finally, the use of molecular methods for the identification and serotyping of *S. pneumoniae* in polymicrobial samples, such as OP specimens, carries a risk of cross-reactivity with commensal streptococcal species that share closely related DNA sequences [45]. To improve specificity, we employed a dual-target qPCR assay targeting both *lytA* and *piaB* [31]. As shown in Supplementary Figure 1, *lytA* and *piaB* Ct values were strongly correlated in NP samples. However, variability in Ct values was observed between the 2 targets in OP samples, which may reflect amplification of nonpneumococcal DNA. NP specimens are generally considered less susceptible to interference from nonpneumococcal streptococci than OP specimens [46]. To further minimize the risk of cross-reactivity in the conventional PCR assays, serogroups/types detected in nonpneumococcal controls were excluded from the analysis. Nevertheless, residual cross-reactivity cannot be entirely excluded, since homologous *cps* loci have been described in commensal oral streptococci and may not be fully accounted for by the methodological controls applied in this study.

## CONCLUSION

We found evidence of older adults being carriers of a wide range of IPD-causing serotypes, although with a lower prevalence than reported in children. Non-PCV13 type pneumococci dominated in both carriage and IPD incidence in recent years. Our results support that contact with young children is one of the main risk factors in older adults for pneumococcal carriage, even in a country with a high uptake of PCVs among children. Further, PPV23 immunization did not seem to affect pneumococcal colonization in older adults. Immunization with higher valency conjugated vaccines is likely required to reduce residual pneumococcal disease among older adults.

## Supplementary Material

ofag452_Supplementary_Data

## Supplementary Data

Supplementary materials are available at *Open Forum Infectious Diseases* online. Consisting of data provided by the authors to benefit the reader, the posted materials are not copyedited and are the sole responsibility of the authors, so questions or comments should be addressed to the corresponding author.
