# Supplementary material for: Prevalence, Serotype Distribution, and Risk Factors Associated With Streptococcus pneumoniae Carriage in Older Adults in Denmark, 2019–2022
Source: Open Forum Infect Dis. 2026 Jul 24;13(8):ofag452. doi: 10.1093/ofid/ofag452 (PMC13435622; doi:10.1093/ofid/ofag452)
Supplement: Supplementary file 1 [file ofag452_supplementary_data.docx]

### Supplementary Table 1. Multiplex PCR serotyping scheme

| **Reaction** | **Serotypes included** | | | | |
| --- | --- | --- | --- | --- | --- |
| Multiplex 1 | 1 | 3 | 4 | 7F/7A | 9V/9A |
| Multiplex 2 | 14 | 6A/6B | 19F | 23F | 19A |
| Multiplex 3 | 8 | 5 | 11A/11D | 20 | 22F/22A |
| Multiplex 4 | 24A/24B/24F | 2 | 12F/12A/44/46 | 9N/9L | 10A |
| Multiplex 5 | 23B | 33F/33A/37 | 34 | 18A/18B/18C/18F | 23A |
| Multiplex 6 | 39 | 7C | 15A/15F | 38/25F/25A | 16F |
| Multiplex 7 | 7C/7B/40 |  | 15B/15C | 35F/47F | 31 |
| Multiplex 8 | 35A |  |  | 17F | 35B |

### Supplementary Table 2. Associations between carriage compared to non-carriage among N=1628 adults

|  | Carriage vs. non-carriage | | | | | |
| --- | --- | --- | --- | --- | --- | --- |
|  | Crude OR | 95% CI | p-value | Adjusted OR | 95% CI | p-value |
| Age |  |  |  |  |  |  |
| 64-69 | ref | - | - | ref ^a^ | - | - |
| 70-74 | 0.9 | 0.6-1.3 | 0.61 | 0.9 | 0.6-1.4 | 0.78 |
| 75-79 | 0.6 | 0.4-1.0 | **0.0347** | 0.7 | 0.4-1.1 | 0.08 |
| >80 | 0.8 | 0.5-1.2 | 0.25 | 0.8 | 0.5-1.4 | 0.48 |
| Sex |  |  |  |  |  |  |
| Female | ref | - | - | ref | - | - |
| Male | 1.0 | 0.7-1.4 | 0.83 | 1.0 | 0.7-1.4 | 0.83 |
| Period of sampling |  |  |  |  |  |  |
| Pre-lockdown | ref | - | - | ref ^b^ | - | **-** |
| Lockdown | 0.3 | 0.2-0.5 | **0.0004** | 0.4 | 0.2-0.7 | **0.0042** |
| Post-lockdown | 0.7 | 0.5-1.0 | 0.06 | 0.9 | 0.6-1.5 | 0.81 |
| Smoking status |  |  |  |  |  |  |
| Non-smoker | ref | - | - | ref ^c^ | - | - |
| Smoker | 0.8 | 0.5-1.4 | 0.49 | 0.9 | 0.5-1.4 | 0.52 |
| Comorbidity |  |  |  |  |  |  |
| No | ref | - | - | ref ^d^ | - | - |
| Yes | 0.8 | 0.5-1.1 | 0.10 | 0.8 | 0.6-1.1 | 0.22 |
| Prior pneumonia |  |  |  |  |  |  |
| No | ref | - | - | ref ^e^ | - | - |
| Yes | 0.7 | 0.5-1.0 | **0.0418** | 0.7 | 0.5-1.1 | 0.09 |
| Antibiotics past 6 months |  |  |  |  |  |  |
| No | ref | - | - | ref ^f^ | - | - |
| Yes | 0.5 | 0.3-1.0 | **0.0464** | 0.6 | 0.3-1.1 | 0.09 |
| Pneumococcal vaccination status |  |  |  |  |  |  |
| None | ref | - | - | ref ^g^ | - | - |
| PCV13 | 1.3 | 0.4-4.4 | 0.71 | 1.3 | 0.4-4.5 | 0.71 |
| PPV23 | 0.7 | 0.5-1.0 | **0.0481** | 0.8 | 0.5-1.3 | 0.33 |
| PCV13 + PPV23 | 0.5 | 0.2-1.0 | **0.0410** | 0.6 | 0.3-1.3 | 0.17 |
| Grandchildren less than 5 y/o |  |  |  |  |  |  |
| No | ref | - | - | ref ^h^ | - | - |
| Yes | 1.8 | 1.3-2.6 | **0.0008** | 1.8 | 1.2-2.5 | **0.0031** |
| Travel abroad past 6 months |  |  |  |  |  |  |
| No | ref | - | - | ref ^i^ | - | - |
| Yes | 1.2 | 0.9-1.7 | 0.23 | 1.0 | 0.7-1.4 | 0.92 |

^a^ Adjusted for sex, comorbidity, pneumoniae, and grandchildren. ^b^ Adjusted for age, comorbidity, and vaccines. ^c^ Adjusted for age, sex, comorbidity, pneumoniae, antibiotics, and vaccines. ^d^ Adjusted for age, sex, smoking, pneumoniae, antibiotics, and vaccines. ^e^ Adjusted for age, comorbidity, antibiotics, and vaccines. ^f^ Adjusted for age, pneumoniae, comorbidity, vaccines, and travel. ^g^ Adjusted for age, period, pneumoniae, and comorbidity. ^h^ Adjusted for age and comorbidity. ^i^ Adjusted for age, comorbidity, and period.

**Supplementary Figure 1. Scatterplots of lytA and piaB CT values in nasopharyngeal (NP) and oropharyngeal (OP) samples**

NP	OP

### Supplementary Figure 2. Distribution of serogroups/-types of carried pneumococci with excluded serogroups-/types detected in non-pneumococcal controls (shaded boxes)
